# Family and healthcare professionals’ perceptions of a pilot hospice at home programme for children: a qualitative study

**DOI:** 10.1186/s12904-016-0161-0

**Published:** 2016-10-28

**Authors:** Maria Brenner, Michael Connolly, Des Cawley, Frances Howlin, Jay Berry, Claire Quinn

**Affiliations:** 1 School of Nursing and Midwifery, Trinity College Dublin, 24 D’Olier Street,, Dublin 2, Ireland; 2 School of Nursing, Midwifery and Health Systems, University College Dublin, Dublin, Ireland; 3 Department of Nursing and Health Science, Athlone Institute of Technology, Athlone, Ireland; 4 Department of Medicine and Division of General Pediatrics, Boston Children’s Hospital and Harvard Medical School, Boston, MA USA; 5 National University of Ireland, Galway, Ireland

**Keywords:** Hospice and palliative care nursing, Child, Family, Qualitative research

## Abstract

**Background:**

Parents commonly report a significant improvement in quality of life following the provision of hospice and supportive care and have identified a need for such a service in the home. The purpose of this study was to understand the experiences of families receiving a nurse led pilot hospice at home programme and the experiences of healthcare professionals delivering and engaging with the programme.

**Methods:**

An exploratory, qualitative study was conducted, including telephone interviews with parents and focus groups and individual interviews with healthcare professionals. All parents of families who received the programme of care between June 2014 and September 2015 and healthcare professionals delivering and engaging with the programme were invited to participate.

**Results:**

Seven parents participated in telephone interviews. Four focus groups took place, two with external stakeholders (18 participants in total), one with in-patient hospice staff (13 participants) and one with the hospice at home team (8 participants). Two additional interviews took place with individual stakeholders who were unable to attend a scheduled focus group. Themes from interviews with parents focused on the value of having consistent and expert care. The findings from healthcare professionals centred on communication within and across services, education and training and lone working.

**Conclusions:**

The pilot hospice at home programme was welcomed by all those who took part in the study. The programme may be improved by enhanced clarification of roles, enhanced access to multi-disciplinary services, greater communication across services and improved information provision to families.

## Background

There has been an increase in the number of children living with life-threatening conditions, in part owing to technological advances and medical progress, meaning that access to palliative care services is required across extended years [1]. In Ireland, recent evidence suggests that there are at least 3840 children living with a life-limiting condition [2]. Parents generally become expert in the clinical care of their child during an acute episode of care, however, there is evidence of burn-out within a short period of time following the transition to becoming the child’s primary care giver, with the potential for physical or mental ill health [3, 4]. Such caregivers report a significant improvement in psychological adjustment, fatigue and mental health quality of life following the provision of respite care [1]. This refers to the provision of care to children with a life-threatening condition for a specific period of time with the intent of providing temporary relief to the main carers and their family. However, there can be challenges in the time it takes to access such services [5] and parents have identified a need for the provision of this service in the home [6, 7]. In addition to international support for the provision of respite care [8, 9] this need was also identified in a number of national reports on palliative care service provision in Ireland. The need for the provision of respite care, as close to the child’s home as possible, was initially acknowledged nationally in *The Report of the National Advisory Committee on Palliative Care* [10], followed by *A Palliative Care Needs Assessment for Children* [11] and *Palliative Care for Children with Life-limiting Conditions in Ireland - A National Policy* [12]. Collectively they recommended the need for relevant structures to support a respite at home service including adequate homecare and community support services, education, training and development of healthcare professionals and enhanced bereavement support structures. A critique of this approach, within the wider international context was previously published [13], a distinctive feature of which is the dependency on registered nurses.

Within this context a nurse led pilot hospice at home programme, delivering respite care to children with a life-threatening condition, was established in 2014 in two regional sites by a children’s hospice. The proposed role of the hospice team included: emotional and social support; provision of information, support, education and training where needed to all carers; 24 h end-of-life care; and bereavement support and respite in the home. It was anticipated that this new service would be expanded. However, as this was the first time such a service was provided in the context of care delivery in the Republic of Ireland it was important to understand the experiences of all stakeholders involved in the delivery and receipt of care, to inform expansion of services that would address the needs of these stakeholders. The aim was our study therefore was to evaluate this pilot service. The specific objectives of the evaluation were: to understand the experiences of the families receiving this service from the perspective of parents; to understand what was working well for those delivering the services and for internal and external stakeholders who engage with the service; and to identify any changes that may be required to enhance the care of the children and their families. The study report follows consolidated criteria for reporting qualitative research (COREQ) [14].

## Methods

A qualitative study was conducted, informed by qualitative evaluation methodology [15]. This approach, based on the principle of relativism, sought to explore the multiple realities of experience of this pilot hospice at home programme from a wide variety of stakeholders. A comprehensive design was put in place to capture the data including telephone interviews, focus groups and individual interviews between February and September 2015. The study was approved by the Research Ethics Committee of the children’s hospice and the associated university. As hospice care for children is a very small and identifiable group in Ireland, in terms of both the children and healthcare professionals, the ethics committee did not permit us to gather demographic detail on the participants.

### Recruitment procedures

Purposive sampling was used to identify parents for participation in the interviews. All parents of families who received the service between June 2014 and September 2015 were invited to participate in an in-depth telephone interview to explore their experiences of the hospice at home programme. In order to minimize family burden and distress, parents of imminently dying children or parents of recently deceased children were not invited to participate. Our decision to interview parents by phone was based on our collective experience of collecting data with vulnerable populations [16, 17] and our acknowledgement of the fact that the parents we sought to speak with often struggled to get time for their own family life. Thirty-seven parents/guardians, who received care from the hospice at home team were invited to participate in the study.

All members of the hospice at home team, including staff in the in-patient hospice service who were linked to the service and external stakeholders who engaged with the hospice at home team (multi-disciplinary stakeholders from a wide variety of linked services including specialist nursing services, medicine and allied healthcare professionals) were invited to participate in focus groups. Through facilitation, focus groups explore cultural and social dynamics in-depth and in a time-efficient way, through engaging with a homogenous group who hold a similar interest, but varied perspectives, on the topic [18].

All potential participants were provided with detailed written information on the aim, purpose and significance of the study and the approximate time commitment required for the interview. Prospective participants were advised that participation was voluntary and they were encouraged to contact one of the researchers if they had any queries or would like to take part in the study. Each person who expressed an interest in participation was contacted by a researcher on up to two occasions to set up an interview time, through the participants chosen form of communication (phone or email).

### Interview protocols

During interviews parents were asked to recall their experience of care from the hospice at home programme, any challenges they encountered, and any recommendations they had for the improvement of the programme (Table 1). Each member of the data collection team had over 20 years’ experience nursing, particularly with children and parents at critical junctures in care delivery. Parents were aware of this and the researchers used their experience to skilfully listen for cues to support parents telling their story. Parents were also informed of the supports in place in place for them should they become upset after the interview. Parents were aware that they could choose to stop the interview at any point without any impact on their access to hospice care delivery.

**Table 1:** Interview schedules

Interview schedule parent interviews^a^	
1. Can you tell me about your experience of care from the hospice at home team? 2. What was the best thing about the programme for your child and family? 3. Can you tell me about any challenges you encountered with the programme? 3. Can you tell me about any changes you would recommend that could enhance your experience of the hospice at home programme?	
Interview schedule focus groups^b^	
1. What is the nature of your role with the hospice at home team? 2. What were your expectations of your role in this programme at the start? 3. How do you see your role at the moment? 3. Are there any local policies and guidance for your interactions with the service? 4. What do you think is working well in the current programme? 5. Can you tell me about any changes you would recommend that could improve the programme?	

^a^interviews conducted with parents

^b^focus groups conducted with external stakeholders, in-patient hospice staff and the hospice at home team

The venue chosen for all focus group interviews was a quiet private room on the grounds of the children’s hospice. Following introductions participants were offered an opportunity to ask any questions about the research process and were asked to sign a consent form to participate in the study. To encourage interaction group participants sat in a close circle around a table, with a number placed in front of each person. Skilled facilitation allowed each participant to engage and to express their opinions and experiences, and to explore and debate opinions expressed by others.

### Analysis

A qualitative descriptive approach was used to analyse the data; it was not the intention to generate a theory or to recontexualise the experience of the parents or healthcare professional participants. The data was transcribed verbatim and the data for each group was initially analysed separately. MC coded the data from each group and identified initial themes. MB, MC, DC and FH reviewed the codes and themes and reconciled differences achieving greater than 90 % agreement. The group then identified similarities and differences in the key themes across all participant groups [19–21]. Themes identified from interviews with parents were not found to be substantially similar to those from interviews with healthcare professionals, while similarities were found among key themes which emerged across the interview data from all healthcare professionals.

Rigor for the study was established using core principles applied within qualitative research [22]. Credibility of the data was established by the inclusion of parents who had experience of receiving care from the hospice at home team and healthcare professionals who delivered this service or were affiliated to it; this also addressed the criterion of authenticity, as an understanding of the constructs of others was sought through evidence of a variety of realities contained in the data collected. Accuracy of the data collected was established through the verbatim transcripts of the audio files, while confirmability of the data was supported by keeping an audit trail of conclusions reached. Transferability of data from the interviews was established by presenting detailed information on sampling, data gathering, analysis and interpretation when reporting the findings [23].

## Results

### Participants

Seven parents participated in the telephone interviews. Four focus groups took place, two with external stakeholders (18 participants in total), one with in-patient hospice staff (13 participants) and one with the hospice at home team (8 participants). Two additional interviews took place with individual stakeholders who were unable to attend a scheduled focus group but who were interested in contributing to the study.

### Themes – parent interviews

Four key themes emerged: expert care ‘at this time in their life’; benefitting the whole family; consistent care; and progressing the service.

#### Expert care ‘at this time in their life’

For parents, caring for a sick child with a life-threatening condition is a full-time and often complex job. Indeed family carers have been described as the ‘conductor’ of care management [24], and are likely to be linked in with multiple service providers and agencies. For parents the availability of experts in the delivery of respite care programme, under the auspices of a wider hospice service, was seen as both important and significant in the context of the care that they felt their child needed. They explained ‘this time in their life’ as their current period of being a primary care giver, delivering 24 h care in the home to a child with multiple care needs and they specified their need at this time for intensive emotional support, and respite from this by competent care delivery in the home. Parents repeatedly referred to the fact that their child had an unpredictable trajectory and they deeply valued how the hospice at home staff understood the importance of this time for them and their child.

… it is extremely good to be able to stay at home and to have your child at home and have that respite at home. I would much prefer it than having to go anywhere… that is the big one, having him at home, being able to keep him at home with everybody … to be able to have him here and to have the break here… having him at home is the key (P3)

Parents identified that this level of holistic care was exceptional in comparison to care they received from non-hospice trained staff. In addition to the expert clinical care that was required at such a fragile and tenuous point in their child’s life, they also greatly valued the attention paid to the child as an individual and highlighted the team’s capacity to hear them and to address the individual needs of their child. Through this they, as a family, felt very cared for at this important time in their lives.

I have seen the two sides, I have seen someone come into my home not understanding how important this time is… then I have had the very positive experience of where these various members of LauraLynn came…They take on every aspect of his care…they have listened to me, they have gone with my son, with whatever he wants to do. But I never felt, I have never once felt that this was a chore for them, that this was a job that they were on the clock. It was something really from their hearts, that they wanted to do, that they wanted to give to my son, not even to me, to my son, that they really cared about him. It is not often you get people like that … LauraLynn is a totally different experience (P1)

#### Benefitting the whole family

A key issue that arose for parents interviewed was the benefit that the hospice at home team provided for the entire family. This was evident in the time available for parents to attend to their own needs or the needs of other children in the family.

…allows you [to] get time with the other children, time to really concentrate on them for a change (P7)

#### Consistent care

For parents of children with a life-threatening condition a major concern is having appropriately qualified nurses looking after their child. The fact that the same person was continually involved in care was a significant positive for parents interviewed, as it allowed for continuity of care and enabled the carer to get to know the child and the family.

…the consistency rather than the ad hoc piece that we get with other services…that is the best bit, the same people that get to know you and your family and follow up with care every week (P5)

#### Progressing the programme

Some parents were keen to point out elements of the hospice at home programme that could be improved. One of these elements related to the provision of written information.

…we don’t get that much written information from any of the people that we are linked in with…It would be nice to have some written information (P2)

Parents also suggested the need for improved notice of when care would occur. This was considered essential in a household that may be linked to several service providers. There was a suggestion that the provision of a two week schedule in advance would be really helpful.

…at the moment it is one week’s notice, but you know the way we have therapies and stuff going on, there is lots of stuff going on so I think 2 weeks’ notice, if they did the schedule 2 weeks in advance (P4)

### Themes – healthcare professionals

All healthcare professionals interviewed welcomed the establishment of the hospice at home programme and supported the need for such a service in the overall provision of care to children with a life-threatening condition and their families. Three key themes emerged across all interviews: communication within and across services, education and training and lone working.

#### Communication within and across services

External stakeholders identified the value of the use of some shared notes to record interactions and the care of the child and their family. This record, called ‘My Story’, was a folder that was kept with the child and served to ensure that details regarding the child’s condition and their care needs were documented. They identified this as helpful in reducing some of the stress on parents having to constantly ‘go over’ their story. However, they also indicated that, in some cases, information pertaining to care of the child and their family was shared between various stakeholder bodies on an ad-hoc basis.

You might ring them, leave a message. They might ring you back, you are on a corridor you’re trying to go somewhere where you can speak freely…it is a safety issue and it is wrong that you are discussing emotive pieces when you are shouting into a phone speaker. (FG2)

The need for enhanced communication and documentation to support continuity of care was also identified between the hospice at home team and by those providing in-patient care in the service.

It is very easy, with the best will in the world, to have verbal conversations…you have to have some mechanism for making sure that all care and communication is documented. (FG3)

While working towards continuity of care within and across services some participants highlighted the need and value of being able to offer a flexible service to children and their families in the home. However, there was concern that flexibility in the delivery of some clinical care practices had the potential to compromise the delivery of safe care.

…we are trying to take it on a case-by-case basis and we have asked everybody to write out their observations and put it through risk …we have been combining those and trending them (FG3).

An important point of concern related to the availability and provision of bereavement support and services to families, in particular those families who were cared for solely by the hospice at home team, who had never been admitted to the in-patient hospice service. Participants stated that the families of children admitted as an in-patient had access to bereavement support when needed, though families of children cared for at home may not have access to these services. This was attributed to limited local availability of bereavement support in the community and lack of clear guidelines and protocols for how this support could be provided from the hospice organisation as a whole.

#### Education and training

The provision of care by nurses, with expertise in caring for children with life-limiting conditions, was very much valued. Some participants had an expectation of highly skilled children’s nurses delivering end-of-life specialist care as part of the hospice at home service. This included symptom management, use of syringe pumps, change of a subcutaneous site and use of a central line while others had an expectation that this would not be part of this service. This, therefore, led to some discussion within each focus group about the minimum training initially required and whether this should be a short theory course or a course that included some practical experience. There was also discussion about the need for ongoing professional development and training for staff, and how this might roll out in the future. For example, if it would include rotation across the hospice services or be confined to day-release training events.

I think we do need to have the processes in place to make sure that we have consistency and continuity…it was quite easy at the beginning where they all started together, it was quite easy to have a focus on them. But as new members of the team are starting we need to make sure that everybody is getting the same consistent induction access to education and training. (FG3)

### Lone working

Participants in the focus group with the hospice at home team identified their role in this pilot hospice at home programme as both stressful and demanding at times, and were very positive in respect of coping and feeling supported in their work. A particular point was made regarding the collegial support that is provided in light of the reality of working in the community environment, particularly working alone where staff have to drive long distances going from one stressed family to another.

… you have got the support of your team member behind you. It is only a phone call away if there is something that you are unhappy about or something that you need to tease out, support is there…there is great support around us. (FG4)

## Discussion

Two key areas emerged across all of the interviews with stakeholders: communication and competency. Parents identified two key communication areas that could enhance their experience of care in the home: written information to support them repeating some of the play and distraction techniques that were used during visits, and more notice of the timing of the respite care so they could make optimum use of that time for the wider family. This is supported in recent research, exploring how well the palliative care needs of children with life-threatening conditions and their families were being met, identifying the need to place continued emphasis on improving the quality and quantity of information for families, with clarity about whose role it is to provide the required information and in guidelines and charters for end of life care for children and their families [25–27]. This issue of communication was also threaded through the findings from healthcare professionals. Specifically the healthcare professionals interviewed focused on the communication of care within and across services providing care to families of children with life-threatening conditions in the home and was part of a wider discourse on the need for all services to be viewed as a sum of the parts rather than parts of the sum. This included concern about equity of access for families to bereavement services, which was previously identified as a core need for these families [7, 9]. The need for a seamless service is supported in literature on parents who reported being stressed when repeatedly asked the same questions about their child’s health within the same service, which can lead to parents becoming more anxious about their child [28]. Communication at the point of the initial referral and ongoing communication pathways, primarily regarding the documentation and handover of care, was identified as a challenge. These challenges are well recognised in literature on respite care and in wider literature on documentation and safe and effective handover practices [28–30]. The point at which a patient’s care is handed over, from one healthcare professional to another, carries inherent risks to patient safety [31]. Informal communications about care needs, via voice mail and telephone, have the potential to go undocumented, which may lead to unintended consequences, such as ‘oversimplification’ or misinterpretation of communicated information [32]. Given the variety of statutory and non-governmental agencies providing care in the home the challenge is to identify a new tool that will fit with the existing workflow processes. This also has the potential to enhance competent care delivery which was also a convergent theme across interviews with parents and healthcare professionals.

Parents interviewed stated that they valued the provision of specialist care in the home, continuity of care and the individualised care they and their child received. This finding is supported in previous research in this area which identified that the most desirable attributes of such staff include compassion, clinical precision and expertise, commitment to the child and family and taking time to discuss a child’s care with the family [28, 33–35]. The individualised care described by the parents in this study included the hospice team’s ability to listen to parents’ wishes about their child’s care and the importance the hospice team placed on understanding individual routines and needs. This supports previous research in this area, which identified these as specific needs of parents of children with complex care needs [36, 37]. The value of having scheduled times for home visits is also supported, however, it is the specialised competency of staff which enhances the quality of life for both the child and family as opposed to the schedule of delivery [33]. It is therefore important to consider the staff skill mix in the scheduling of visits in order to meet the specific requirements of parents in the delivery of homecare [30]. Developing and maintaining staff competency was identified as a challenge in the Irish setting as some healthcare professionals identified variations in clinical care practices across the inpatient hospice setting and the homecare environment. This led to discussion of education and training requirements with little consensus reached on what they should be for those delivering hospice care in the home in this service. However, this problem is not unique to the Irish setting. It is consistent with the diverse approach to the education and preparation of nurses and healthcare professionals who care for these children and their families across Europe and a distinct lack of consensus regarding the specific education requirements to do so. It is acknowledged that the organisation of care for children with life-threatening conditions in the community in Ireland is in a significant development phase, and there is a need for agreement, direction and guidance. However, the *Report of the European Association for Palliative Care Children’s Palliative Care Education Taskforce* [38] outlined core competencies for education in children’s palliative care, while in Ireland the *Palliative Care Competence Framework* [39] provides for core competences in palliative care whilst also detailing individual competences for health and social care professionals from a variety of disciplines, working in various clinical settings and at various different levels.

### Limitations

A number of parents who initially expressed interest in participating did not subsequently present for interview. It is acknowledged that this may be for a variety of reasons including the demands of being a primary care giver for a child with a life-threatening illness and research fatigue [40]. The use of telephone interviews as opposed to face-to-face interviews could be viewed as a limitation of this study. Some qualitative methodologists are not in favour of this approach suggesting that telephone interviews may be used as a time-saving exercise and that it may hinder the researcher’s ability to build a rapport with the participants and thereby hinder the opportunity to gather in-depth data [41, 42]. This was not our experience. Our decision to interview parents by phone was based on our collective experience of collecting data with vulnerable populations and our acknowledgement of the fact that the parents we sought to speak with often struggled to get time for their own family life. Therefore we deliberately sought an approach that would not impose on them any more than was necessary.

## Conclusions

The hospice at home pilot programme was welcomed by all those who took part in the study. However, tangible issues that could be addressed to support the continuation and further development of the programme include: further clarification of roles; exploration of access to multi-disciplinary support services that are currently available for the families of children who receive in-house hospice care; enhanced communication across services; consistent and continuous staff education; and improved information provision to families.
